# Dosimetric characterizations of GZP6 ^60^Co high dose rate brachytherapy sources: application of superimposition method

**DOI:** 10.2478/v10019-012-0005-3

**Published:** 2012-01-02

**Authors:** Mohammad Taghi Bahreyni Toossi, Mahdi Ghorbani, Ali Asghar Mowlavi, Ali Soleimani Meigooni

**Affiliations:** 1 Medical Physics Research Center, Medical Physics Department, Faculty of Medicine, Mashhad University of Medical Sciences, Mashhad, Iran; 2 North Khorasan University of Medical Sciences, Bojnurd, Iran; 3 Physics Department, School of Sciences, Hakim Sabzevari University, Sabzevar, Iran; 4 Comprehensive Cancer Center of Nevada, 3730 S. Eastern Avenue, Las Vegas, Nevada, USA

**Keywords:** brachytherapy, GZP6, TG-43, superimposition method, Monte Carlo simulation

## Abstract

**Background:**

Dosimetric characteristics of a high dose rate (HDR) GZP6 Co-60 brachytherapy source have been evaluated following American Association of Physicists in MedicineTask Group 43U1 (AAPM TG-43U1) recommendations for their clinical applications.

**Materials and methods:**

MCNP-4C and MCNPX Monte Carlo codes were utilized to calculate dose rate constant, two dimensional (2D) dose distribution, radial dose function and 2D anisotropy function of the source. These parameters of this source are compared with the available data for Ralstron ^60^Co and microSelectron^192^Ir sources. Besides, a superimposition method was developed to extend the obtained results for the GZP6 source No. 3 to other GZP6 sources.

**Results:**

The simulated value for dose rate constant for GZP6 source was 1.104±0.03 cGyh-1U-1. The graphical and tabulated radial dose function and 2D anisotropy function of this source are presented here. The results of these investigations show that the dosimetric parameters of GZP6 source are comparable to those for the Ralstron source. While dose rate constant for the two ^60^Co sources are similar to that for the microSelectron^192^Ir source, there are differences between radial dose function and anisotropy functions. Radial dose function of the ^192^Ir source is less steep than both ^60^Co source models. In addition, the ^60^Co sources are showing more isotropic dose distribution than the ^192^Ir source.

**Conclusions:**

The superimposition method is applicable to produce dose distributions for other source arrangements from the dose distribution of a single source. The calculated dosimetric quantities of this new source can be introduced as input data to the GZP6 treatment planning system (TPS) and to validate the performance of the TPS.

## Introduction

Recently, the GZP6 high dose rate (HDR) ^60^Co brachytherapy unit, manufactured byNuclear Power Institute of China (Chengdu, China), has been introduced for brachytherapy procedures.^1^ Although not as common as ^192^Ir source, ^60^Co has been used in past as a brachytherapy source in the treatment of various malignancies^2^–^4^, and a brachytherapy technique remains a frequent treatment in clinical praxis.^5^ There are also potential logistical advantages for ^60^Co sources for HDR systems over ^192^Ir^6^, including less frequency of source exchange which provides longer duration of clinical use and reduced operating costs. Different geometric designs of ^60^Co radionuclide have been used in radiotherapy clinics in the past with limited traditional dosimetric information.^7^,^8^ Dose calculations around these implants were performed using the traditional dose calculation technique. However, presently, the Task Group 43 (TG-43) report of American Association of Physicists in Medicine(AAPM) has recommended determination of dosimetric parameters, such as radial dose function, anisotropy function (ANF), etc., of any brachytherapy sourcebefore its clinical use.^9^ Also according to the AAPM TG-56 such parameters are required as input data and for verification of the treatment planning system.^10^

**FIGURE 1:**
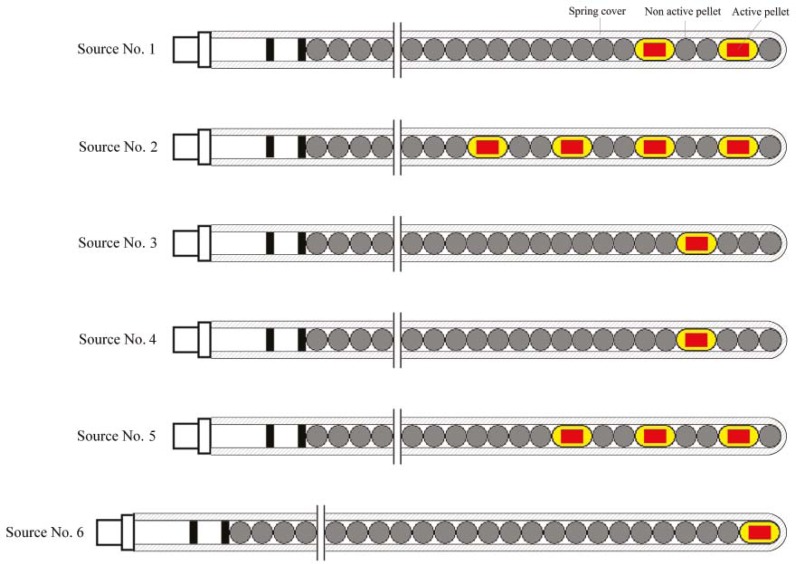
Schematic diagram illustrating six GZP6 source braids, containing active and nonactive pellets. Each source is allocated to one separate channel. The sources 1–5 are stationary while the source No. 6 is stepper.

**FIGURE 2:**
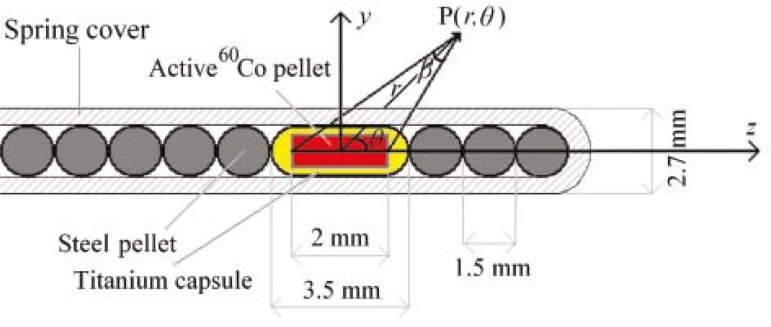
A schematic view of the GZP6 source braid number 3 illustrating the dimensions of active cylindrical ^60^Co and non-active steel pellets as well as TG43U1 coordinate systems. This diagram is schematic and is not to scale.

The newly designed GZP6 HDR ^60^Co brachytherapy unit has been recently employed for the clinical practice in Iran. Unlike the HDR ^192^Ir systems, which contain one source, the GZP6 unit includes six different sources (Figure 1). Each source is designated to a separate channel in the HDR unit. Five of these six sources are stationary, and the 6^th^ source (*i.e.* source number 6) has stepping (dwelling) capability that could be used for treatment of patients with longer active length. As shown in Figure 1, each source is composed of a source-braid or packing, consist of 1, 2, 3 or 4 radioactive source pellets as well as a number of non active steel pellets. In order to make these source geometries reproducible, at the same time flexible as it moves within the transfer tube and applicator, the active and non-active pellets are fitted within a steel spring cover. Dimensions and components of the active and non-active pellets of these sources are provided in the schematic diagram of source number 3 (Figure 2). In their first publication on this system, Mesbahi *et al.* measured the air kerma strengthsof source numbers 1, 2 and 5.^11^ In a separate investigation, Mesbahi has also calculated radial dose function for these three sources.^12^ Naseri *et al*. have examined the accuracy of the dose distributions calculated by the GZP6 treatment planning system by Monte Carlo simulation of these sources.^13^ Monte Carlo simulation is widely used in radiophysics^14^, however, to our knowledge the GZP6 unit has not been studied before, based on a comprehensive determination of TG-43U1 dosimetric parameters.^15^ Since the GZP6 unit has six sources with different fixed configurations, each source has been individually evaluated for their clinical applications.

The goal of this project is to investigate the dosimetric parameters (*i.e*. dose rate constant, radial dose function and anisotropy function) of source number 3, which has not been studied before, following the TG-43U1 recommendation through Monte Carlo simulations. The results of these investigations will be compared with the corresponding data available for Ralstron (type 2) ^60^Co and microSelectron ^192^Ir sources.^4^,^16^ The dose distribution for the source No. 3 is used to produce dose distributions for the other GZP6 sources using a superimposition method developed in this study.

## Materials and methods

### Radioactive source structure

The GZP6 high dose rate afterloading unit comprises of different six ^60^Co sources affixed to six different channels in the system. In this study dosimetric characteristic of the source number 3 of the unit, which is a non-stepping source and includes one active cylindrical ^60^Co pellet, is being evaluated. As illustrated schematically in Figure 2, this source braid consists of an active nickel-plated cobalt pellet and a number of inactive spherical pellets which are made of steel. The active ^60^Co cylinder has a radius of 0.5 mm and length of 2 mm (including the nickel-plating), in which the ^60^Co radionuclide was distributed uniformly throughout the core. The nickel plating has a thickness of 0.05 mm, which has not been illustrated in the Figure 2. The active cobalt pellet is additionally encapsulated in a titanium capsule, with 0.25 mm in thickness and consisting of two hemispheres with 0.75 mm in radius at the two ends, sealed by Argon arc welding. The whole pellets are accommodated in a steel spring cover with an external diameter of 2.7 mm. ^60^Co is emitting two gamma photons with 1.17 and 1.33 MeV energies and *β*-particles with *E*_max_=0.318 MeV. It has a half-life of 5.271 years. The latter particle is attenuated effectively with the titanium capsule.

### TG 43 formalism

The formalism of TG-43U1 was followed to compute the dosimetric parameters of the source.^15^ According to this formalism the dose rate at the point P(*r*, *θ*) from the source center is defined by the following equation (as illustrated in Figure 2):

[1] $$D(r,\theta )=S_{k}\Lambda \frac{G(r,\theta )}{G(r_{0},\theta_{0})}g(r,\theta )F(r,\theta )$$

Where *r* is the radial distance (in cm) from the source center and *θ* is the polar angle. *r*_0_ is the reference radial distance of 1 cm and *θ*_0_ is the reference polar angle of 90º. *S_k_* is air kerma strength of the source (in U, where 1 U=1 *μ*Gym^2^h^−1^), Λ is dose rate constant, *G*(*r*,*θ*) is geometry function, *g*(*r*,*θ*) is radial dose function and *F*(*r*,*θ*) is two-dimensional (2D) anisotropy function.

The dose rate constant is the ratio of dose rate at the reference point (*r*_0_,*θ*_0_) and air kerma strength:

[2] $$\Lambda =\frac{\dot{D}(r_{0},\theta_{0})}{S_{k}}$$

Dose rate constant has a unit of cGyh^−1^U^−1^.

In this study the line-source approximation was used for calculation of geometry function. By using this approximation, for the geometry function can be obtained from the following equation:

[3] $$G(r,\theta )={\left(r^{2}-L^{2}/4\right)}^{-1}$$

If *θ* ≠ 0° the geometry function is obtained from equation [4]:

[4] $$G(r,\theta )=\frac{\beta }{Lr\text{sin}\theta }$$

As denoted by Awan *et al.*^17^, considering the source active length *L,* the radial distance *r* and the angles *θ* and *ß* as showed in the Figure 2, equation [5] can be resulted from the above equation:

[5] $$\text{G}(r,\theta )=\frac{{\text{tan}}^{-1}[(r\text{cos}\theta +L/2)/r\text{sin}\theta ]-{\text{tan}}^{-1}[(r\text{cos}\theta -L/2)/r\text{sin}\theta ]}{Lr\text{sin}\theta }$$

Thus if *θ* ≠ 0°, the geometry function can be calculated directly in terms of *r* and *θ* from equation [5].

The radial dose function, *g*(*r*), and anisotropy function, *F*(*r*, *θ*) are defined as:

[6] $$g(r)=\frac{\dot{D}(r,\theta_{0})}{\dot{D}(r_{0},\theta_{0})}\frac{G(r_{0},\theta_{0})}{G(r,\theta_{0})}$$

[7] $$F(r,\theta )=\frac{\dot{D}(r,\theta )}{\dot{D}(r,\theta_{0})}\frac{G(r,\theta_{0})}{)G(r,\theta )}$$

In which *Ḋ*(*r*, *θ*) denotes the dose rate at the point P(*r*, *θ*) from the source.

### Monte Carlo calculations

The MCNP-4C Monte Carlo code was employed to estimate absorbed dose towards obtaining TG-43U1 parameters.^18^ Since it has the option of defining the mesh grids, the MCNPX version 2.4.0 Monte Carlo code was also utilized to obtain 2D dose distribution for the source.^19^ When this option is used it would be easier to score tally values in a large number of scoring volumes. Table 1 lists the mass density and chemical composition of the materials used in our Monte Carlo calculations.

In calculations of dose rate constant and 2D dose distributions, the value of air kerma strength was taken from our previous work.^20^

The source braid was centered in a cylindrical water of dimensions *R*=25 cm and *L*=50 cm in the simulations. A cutoff energy of 10 keV was used for both photons and electrons. To speed up the Monte Carlo calculations, the absorbed dose was approximated as kerma to estimate dose at points where the electronic equilibrium exists. For a ^60^Co source, the dose build up region affects only points in close vicinity from the ^60^Co pellet. The f6 tally was used to score collision kerma (in MeV/g/photon). At the points in the vicinity of the source, in which the electronic equilibrium may not exist, dose was scored using *f8 tally (in MeV/photon). Toroid tally cells of 0.05 cm in thickness were used to score the tally values. The numbers of photon histories simulated were 1.6×10^7^, 1.6×10^7^ and 1.45×10^8^ respectively to obtain dose rate constant, radial dose function, and 2D anisotropy function. The corresponding statistical errors were respectively equal to 1.58, 0.5 and 0.88 percent for the Monte Carlo calculations in the used tally cells. Dose values at different radial distances and angles from the source (required for calculation of radial dose function and anisotropy function) were obtained through Monte Carlo simulations for a water phantom. The data for radial dose function and anisotropy function then were calculated respectively using equations [6] and [7]. Dose rate constant, radial dose function and 2D anisotropy function values for GZP6 source number 3 were compared with corresponding data for Ralstron (type 2) ^60^Co source, reported by Papagiannis *et al.*^4^, and microSelectron ^192^Ir source, reported by Karaiskos *et al..*^16^

To obtain 2D dose rate table, a cylindrical grid of 14 cm×14 cm in *y*-*z* plane, with resolution of 0.05 cm in both longitudinal and radial directions was overlaid on the geometry. The thin grid was used to reduce the Monte Carlo computation time. 3.5×10^8^ photon histories were followed, resulting an average error of 0.44% over the mesh cells. MCNPX tally type 1 with the option of depth for photons was used to determine energy deposition per volume per photon in terms of per photon. The tally value then was converted to the dose rate per U by introducing the activity, air kerma strength of the source and appropriate conversion factors in the calculations.

### Superimposition method

A superimposition algorithm was developed to produce the dose distributions of the GZP6 sources No. 1, 2 and 5 from the dose distribution data of the source No. 3. The algorithm was consisted of four steps:

- The dose distribution data for each pellet in the sources 1, 2 and 5 was obtained from the dose distribution matrix of source 3 through application of matrix shift method.^21^ The shifting distance was equal to the inter-pellets distance (6.5 mm).
- The dose distribution data for each pellet was multiplied by normalized activity of that pellet to the activity of source No. 3.
- A summation was then performed over the matrixes of all pellets in each source of 1, 2 ad 5.
- Dose contours were plotted from the dose distribution matrix of sources 1, 2 and 5.

The results of the mentioned algorithm were verified to examine its accuracy. For this purpose the sources 1, 2 and 5 were simulated separately and the obtained dose distributions were compared by the dose distributions resulted from the above algorithm.

## Results and discussion

### TG-43 dosimetric parameters

The calculated values of geometry function for 0.5–5 cm radial distance and 0°–90° angles are presented in the Table 2. The calculated values for geometry function were equal to unity at 6–10 cm distance. To summarize the results, these values were not presented in the Table 2. The geometry function is symmetrical with reference to the angle of 90°. On the other hand the angular values of the on the either side of the 90° angle, for the angles 100°–180°, are mirror images of the values for 80°-0° angles.^22^ So as showed in Table 2, the two dimensional geometry function is presented only for angles 0°–90°.

Monte Carlo calculated value of air kerma strength is *S*_k=_17240.01 cGyh^−1^U^−1^ as worked out earlier for the GZP6 source number three.^20^ The value was used for the calculation of dose rate constant in the present study. Monte Carlo calculated dose rate constants for GZP6 and Ralstron (type 2) and microSelectron sources are presented in Table 3.

The dose rate results, in terms of cGyh^−1^U^−1^, in Cartesian away (*y*) and along (*z*) coordinates are presented in the Table 4. It should be noted that the source center is assumed to be in the origin of the Cartesian coordinates.

Radial dose function (RDF) computed values for GZP6 source number 3 are presented in Table 5. The tabulated *g*(*r*) data presented in the Table 5 were fitted to a fifth order polynomialin form of:

[8] $$g(r)=a_{5}r^{5}+a_{4}r^{4}+a_{3}r^{3}+a_{2}r^{2}+a_{1}r+a_{0},R^{2}=0.9989$$

The parameters of the fitted polynomial are as follows:

$$\begin{array}{l} a_{5}=1.000\times 10^{-6},a_{4}=-5.000\times 10^{-5},a_{3}=0.0007,a_{2}=-0.0039,\\ a_{1}=-0.01,a_{0}=1.0087. \end{array}$$

**FIGURE 3:**
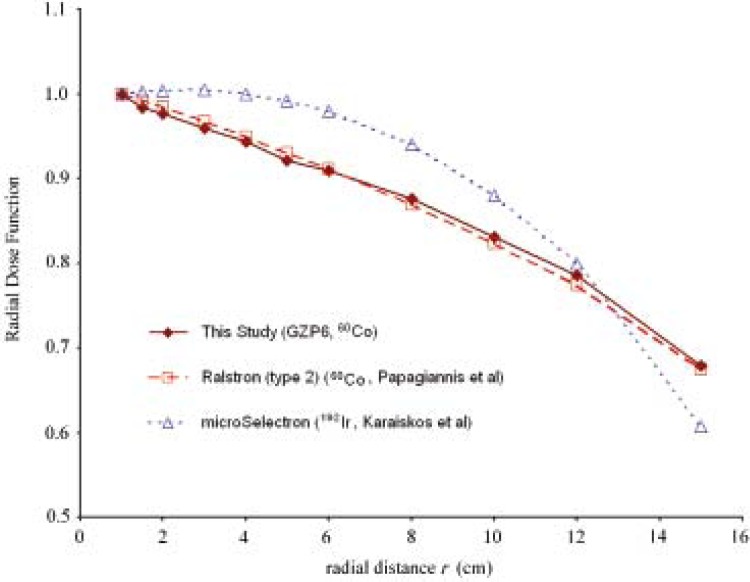
Radial dose function versus radial distance from the source for GZP6 ^60^Co (number 3), Ralstron (type 2) ^60^Co (by Papagiannis *et al*.^4^) and microSelectron ^192^Ir (by Karaiskos *et al*.^16^) sources.

A graphical representation of *g*(*r*) values for the GZP6 source is presented in Figure 3 and compared with the corresponding data for the Ralstron (type 2) ^60^Co source reported by Papagiannis *et al*.^4^ and microSelectron ^192^Ir source reported by Karaiskos *et al..*^16^

**FIGURE 4:**
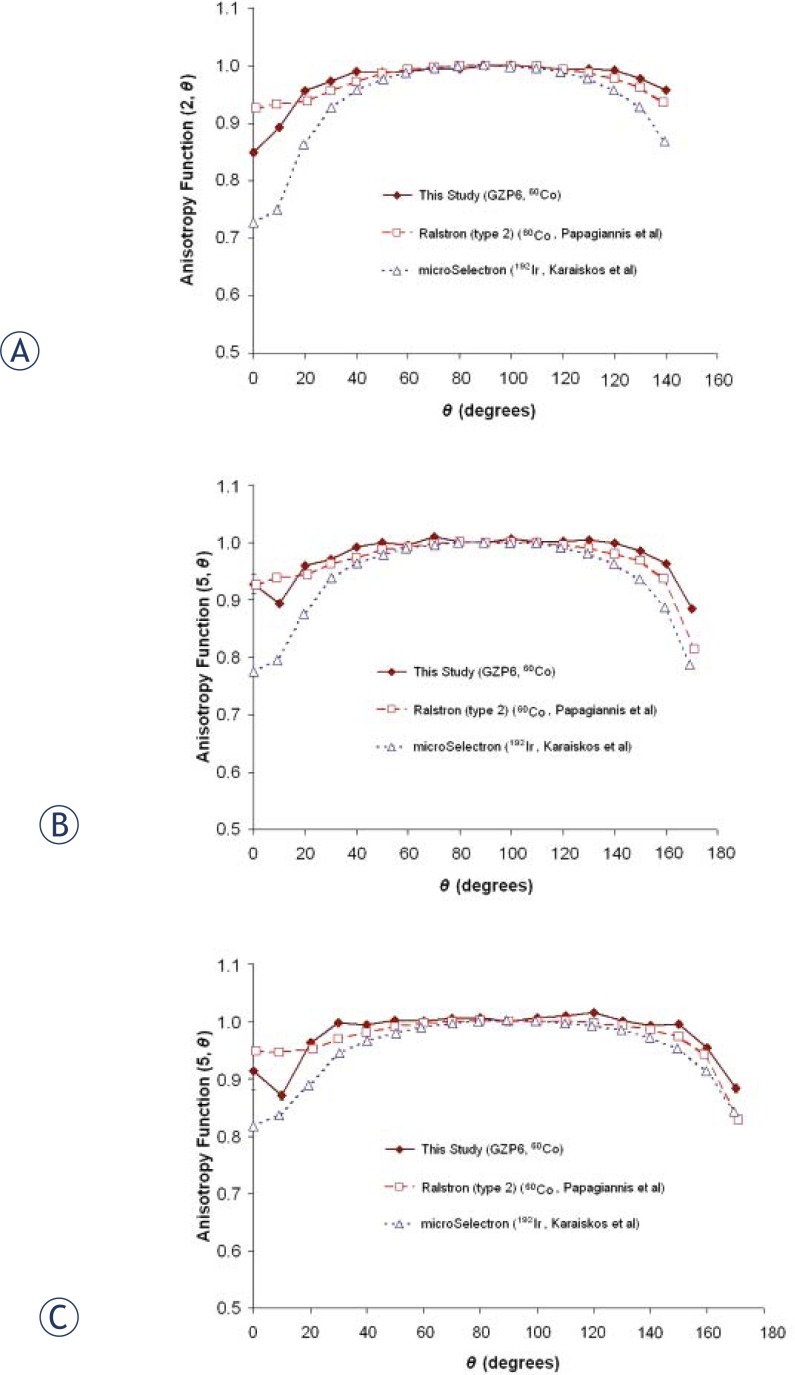
Anisotropy functions of GZP6 ^60^Co (number 3), Ralstron (type 2) ^60^Co (by Papagiannis *et al*.^4^) and microSelectron ^192^Ir (by Karaiskos *et al*.^16^) sources: (A) for *r*=2 cm; (B) for *r*=5 cm; (C) for *r*=10 cm.

Table 6 presents the anisotropy function data for GZP6 ^60^Co source number 3. *F*(*r*, *θ*) values of the GZP6, Ralstron and microSelectron sources for distances of 2, 5 and 10 cm are presented in Figure 4 A–C.

Our results are indicating that the dose rate constant, radial dose function and anisotropy function for GZP6 and Ralstron (type 2) ^60^Co sources are comparable. While the value of dose rate constant for the GZP6 and Ralstron (type 2) ^60^Co sources are close to that for the microSelectron ^192^Ir source (Table 3), radial dose function fall off for the ^192^Ir source is less steep compared to the cobalt sources especially for lower radial distances from the source (in Figure 3). It can be also noticed from the Figure 4 that the two cobalt sources have more isotropic anisotropy functions than the iridium source.

The dosimetric data presented in this study for GZP6 source (number 3) can be utilized in clinical practice of the source and towards the improvement of its treatment planning system.

### Dose distributions from the superimposition method

**FIGURE 5:**
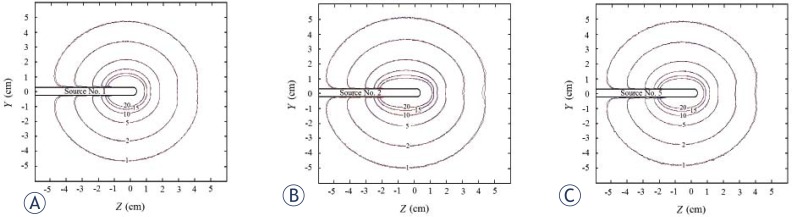
Dose distributions (Gy) obtained by MC simulations (blue lines) and superimposition method (red lines) for GZP6 sources: (a) source No. 1; (b) source No. 2 and (c) source No. 5. Isodoses of 1–20 Gy are contoured in the figure and since the contours by the two methods are overlapped in many points, are almost not distinguishable.

The dose distributions for the GZP6 source No. 1, 2 and 5 which was obtained by MC simulations of the sources and the superimposition method described are presented in the Figure 5. The dose contours of 1, 2, 5, 10, 15 and 20 Gy were plotted. As it can be observed the dose distributions obtained by the two methods for the sources 1, 2 and 5 are equal.

The superimposition algorithm developed in this study is able to produce dose distributions for the GZP6 sources No. 1, 2 and 5 from the dose distribution of the source No. 3. The algorithm is also applicable for other bracytherapy units in which an arrangement of sources is used in the treatment process. These results may provide comprehensive dosimetric information that could be clinically more applicable for dose calculations around different implants by superimposition method.

## Figures and Tables

**TABLE 1:** Mass density and compostion of the materials used in the Monte Carlo simulations

**Material: description**	**Mass density (g/cm^3^)**	**Composition (element/weight fraction)**
Cobalt: source core	8.85	Co/1
Nickel: source plating	8.902	Ni/1
Titanium: source capsule	4.54	Ti/1
Steel pellets: spacers in the source braid	7.9	Fe/0.71994, C/0.0005, Si/0.0072, Mn/0.0137, S/0.00011, P/0.00025, Cr/0.17, Ni/0.0822, Mo/0.0013, V/0.0006, Ti/0.0042
Steel: spring cover	6.999	Fe/0.7416, Ni/0.069, S/0.0001, Cr/0.167, C/0.0006, Mn/0.0062, Cu/0.0026, Al/0.0062, Mo/0.0015, Si/0.0052
Air	0.001205	C/0.000124, N/0.7555267, O/0.231781, Ar/0.012827

Water: phantom material	1	H/0.111894, O/0.888106

**TABLE 2:** Two dimensional geometry function () for GZP6 source number 3

**θ (°)**										
**r(cm)**	**0°**	**10°**	**20°**	**30°**	**40°**	**50°**	**60°**	**70°**	**80°**	**90°**
0.5	1.037	1.036	1.031	1.024	1.016	1.008	1.000	0.994	0.990	0.988
0.75	1.016	1.016	1.014	1.011	1.007	1.003	1.000	0.997	0.995	0.995
1	1.009	1.009	1.008	1.006	1.004	1.002	1.000	0.998	0.997	0.997
1.5	1.004	1.004	1.003	1.003	1.002	1.001	1.000	0.999	0.999	0.999
2	1.002	1.002	1.002	1.002	1.001	1.000	1.000	1.000	0.999	0.999
2.5	1.001	1.001	1.001	1.001	1.001	1.000	1.000	1.000	1.000	1.000
3	1.001	1.001	1.001	1.001	1.000	1.000	1.000	1.000	1.000	1.000
3.5	1.001	1.001	1.001	1.000	1.000	1.000	1.000	1.000	1.000	1.000
4	1.001	1.001	1.000	1.000	1.000	1.000	1.000	1.000	1.000	1.000
4.5	1.000	1.000	1.000	1.000	1.000	1.000	1.000	1.000	1.000	1.000
5	1.000	1.000	1.000	1.000	1.000	1.000	1.000	1.000	1.000	1.000

**TABLE 3:** Dose rate constant (Λ) values for GZP6 ^60^Co (number 3), Ralstron (type 2) ^60^Co (Ref. 4) and microSelectron ^192^Ir (Ref. 16) sources

Source Type	GZP6 (This Study)	Ralstron (type 2) (Papagiannis *et al.*^4^)	microSelectron (Karaiskos *et al*.^16^)
Λ (cGyh^−1^U^−1^)	1.104±0.03	1.101±0.005	1.116±0.006

**TABLE 4:** Dose rate values (in cGyh-1U-1) for GZP6 source number 3

**z (cm)**	**y (cm)**														
	**0**	**0.25**	**0.5**	**0.75**	**1**	**1.5**	**2**	**2.5**	**3**	**4**	**5**	**6**	**8**	**10**	**14**
14	0.00366	0.00348	0.00349	0.00351	0.00355	0.00360	0.00362	0.00364	0.00361	0.00356	0.00346	0.00330	0.00293	0.00251	0.00179
10	0.00776	0.00747	0.00753	0.00762	0.00775	0.00782	0.00783	0.00779	0.00765	0.00731	0.00683	0.00627	0.00512	0.00408	0.00257
8	0.01268	0.01233	0.01242	0.01259	0.01268	0.01272	0.01271	0.01249	0.01216	0.01122	0.01007	0.00893	0.00679	0.00517	0.00301
6	0.02344	0.02289	0.02322	0.02358	0.02372	0.02358	0.02300	0.02212	0.02088	0.01808	0.01534	0.01284	0.00901	0.00640	0.00347
5	0.03438	0.03373	0.03461	0.03494	0.03486	0.03422	0.03282	0.03072	0.02829	0.02343	0.01902	0.01544	0.01029	0.00706	0.00369
4	0.05513	0.05436	0.05559	0.05558	0.05527	0.05295	0.04905	0.04425	0.03936	0.03047	0.02357	0.01838	0.01156	0.00768	0.00387
3	0.10007	0.09995	0.10120	0.10029	0.09802	0.08880	0.07717	0.06576	0.05547	0.03962	0.02874	0.02139	0.01277	0.00825	0.00404
2.5	0.14581	0.14691	0.14702	0.14424	0.13804	0.11895	0.09868	0.08070	0.06597	0.04472	0.03140	0.02291	0.01334	0.00848	0.00411
2	0.23037	0.23371	0.23087	0.22057	0.20321	0.16323	0.12726	0.09901	0.07780	0.04994	0.03406	0.02428	0.01382	0.00869	0.00416
1.5	0.41507	0.42096	0.40432	0.36373	0.31538	0.22747	0.16335	0.11957	0.09008	0.05494	0.03631	0.02549	0.01423	0.00886	0.00421
1	0.96404	0.95030	0.82069	0.65489	0.51070	0.31436	0.20414	0.14060	0.10142	0.05909	0.03812	0.02642	0.01452	0.00897	0.00424
0.75	-	1.64251	1.25332	0.90067	0.64913	0.36206	0.22361	0.14951	0.10613	0.06068	0.03887	0.02673	0.01461	0.00903	0.00426
0.5	-	3.24317	1.98378	1.22459	0.80260	0.40566	0.23943	0.15679	0.10993	0.06193	0.03931	0.02696	0.01469	0.00905	0.00427
0.25	-	7.37207	3.01733	1.55687	0.93343	0.43739	0.25079	0.16130	0.11215	0.06268	0.03962	0.02714	0.01475	0.00907	0.00427
0	-	12.32321	3.63819	1.71023	0.98796	0.44908	0.25446	0.16313	0.11306	0.06297	0.03976	0.02718	0.01481	0.00910	0.00427
−0.25	-	7.37187	3.01661	1.55670	0.93376	0.43736	0.25056	0.16142	0.11212	0.06262	0.03961	0.02716	0.01476	0.00907	0.00427
−0.5	-	3.24423	1.98345	1.22515	0.80214	0.40577	0.23968	0.15661	0.10981	0.06196	0.03935	0.02695	0.01470	0.00905	0.00426
−0.75	-	1.64362	1.25309	0.90124	0.64938	0.36188	0.22361	0.14959	0.10621	0.06073	0.03882	0.02675	0.01466	0.00903	0.00425
−1	-	0.95463	0.82232	0.65543	0.51092	0.31428	0.20416	0.14068	0.10151	0.05911	0.03812	0.02639	0.01452	0.00897	0.00424
−1.5	-	0.41756	0.40609	0.36472	0.31569	0.22760	0.16345	0.11980	0.09005	0.05499	0.03636	0.02548	0.01421	0.00885	0.00420
−2	-	0.22359	0.23240	0.22150	0.20379	0.16334	0.12738	0.09904	0.07777	0.04997	0.03406	0.02431	0.01383	0.00869	0.00416
−2.5	-	0.13517	0.14677	0.14507	0.13834	0.11910	0.09893	0.08086	0.06605	0.04477	0.03147	0.02295	0.01334	0.00849	0.00411
−3	-	0.08853	0.09925	0.10101	0.09848	0.08899	0.07729	0.06578	0.05563	0.03965	0.02878	0.02145	0.01280	0.00825	0.00405
−4	-	0.04431	0.05223	0.05502	0.05557	0.05320	0.04907	0.04429	0.03938	0.03057	0.02360	0.01836	0.01156	0.00768	0.00388
−5	-	0.02532	0.03133	0.03368	0.03451	0.03434	0.03291	0.03077	0.02835	0.02347	0.01907	0.01545	0.01028	0.00706	0.00369
−6	-	0.01587	0.02029	0.02213	0.02306	0.02367	0.02307	0.02216	0.02090	0.01808	0.01534	0.01289	0.00902	0.00641	0.00348
−8	-	0.00756	0.00999	0.01126	0.01192	0.01256	0.01270	0.01251	0.01219	0.01124	0.01008	0.00894	0.00683	0.00516	0.00302
−10	-	0.00423	0.00571	0.00654	0.00701	0.00753	0.00771	0.00777	0.00768	0.00734	0.00683	0.00627	0.00511	0.00408	0.00257
−14	-	0.00183	0.00244	0.00279	0.00305	0.00333	0.00346	0.00355	0.00358	0.00356	0.00347	0.00331	0.00293	0.00251	0.00178

**TABLE 5:** Monte Carlo calculated radial dose functions of GZP6 source number 3

**Radial distance r (cm)**	**g_L_** **(r)**
1	1.000
1.5	0.983
2	0.976
2.5	0.968
3	0.959
3.5	0.950
4	0.943
4.5	0.932
5	0.921
6	0.909
7	0.890
8	0.876
9	0.851
10	0.831
12	0.785
13	0.767
15	0.679
20	0.539

**TABLE 6:** 2D anisotropy functions for GZP6 ^60^Co source (number 3) calculated by Monte Carlo code

**θ (degrees)**	**r (cm)**					
	**1**	**2**	**3**	**4**	**5**	**10**
0	0.858	0.849	0.863	0.904	0.927	0.913
10	…	0.893	0.898	0.901	0.893	0.870
20	0.990	0.956	0.957	0.957	0.959	0.963
30	…	0.973	0.974	0.970	0.970	0.998
40	0.992	0.990	0.988	0.989	0.991	0.994
50	…	0.989	0.986	0.990	0.999	1.002
60	1.011	0.990	0.985	0.982	0.995	1.001
70	…	0.995	1.000	1.002	1.010	1.005
80	1.025	0.994	0.995	0.992	1.001	1.007
90	1.000	1.000	1.000	1.000	1.000	1.000
100	0.995	1.000	1.004	1.004	1.007	1.006
110	…	0.998	1.001	0.999	1.001	1.010
120	0.978	0.993	0.995	0.993	1.001	1.015
130	…	0.995	0.998	0.998	1.004	1.000
140	1.019	0.992	0.992	0.990	0.998	0.993
150	…	0.977	0.979	0.978	0.984	0.995
160	…	0.958	0.958	0.956	0.963	0.954
170	…	…	0.881	0.876	0.884	0.883
